# Biomarkers of Oxidative Stress in Experimental Models and Human Studies with Nutraceuticals: Measurement, Interpretation, and Significance 2017

**DOI:** 10.1155/2017/3457917

**Published:** 2017-08-27

**Authors:** Ilaria Peluso, Maura Palmery, Gregor Drummen

**Affiliations:** ^1^Peluso Research Centre for Food and Nutrition, Council for Agricultural Research and Economics (CREA-AN), Via Ardeatina 546, 00178 Rome, Italy; ^2^Department of Physiology and Pharmacology “V. Erspamer”, “Sapienza” University of Rome, Rome, Italy; ^3^Cellular Stress and Ageing Program, Hepato-Renal Pathobiology Program, Bio&Nano Solutions-Lab3Bio, Bielefeld, Germany

Oxidative stress, the imbalance between reactive oxygen species (ROS) formation and enzymatic and nonenzymatic antioxidants, is involved in the pathogenesis and progression of many ageing-associated diseases, such as cardiovascular disease and certain forms of cancer and neurodegenerative diseases. Many mechanisms and molecular pathways have been implicated in the redox modulation of carcinogenesis and tumor progression. In this context, S. Wang et al. reviewed the involvement of caveolin-1, a constituent protein of caveolae, in cancer promotion and progression, its redox modulation, and its potential role as target of antioxidants. A significant research effort focused on the determination of the antioxidant capacity of natural products and their mode of antioxidant action by using numerous assays and in vitro and animal models. In addition to the antioxidant activity, the modulation of the expression of genes that are regulated by nuclear factor-erythroid 2-related factor 2 (Nrf2), such as antioxidant enzymes, and nuclear factor-kappa B (NF-*κ*B) have been studied extensively; the latter is distinctively involved in immune and inflammatory responses. Many nutraceuticals exert their anti-inflammatory and antioxidant effects through the inhibition of NF-*κ*B and the activation of Nrf2.

K. Anilkumar et al. evaluated the effect of isoorientin isolated from tubers of *Pueraria tuberosa* on lipopolysaccharide- (LPS-) activated RAW 264.7 cells and on mouse paw edema and air pouch models of inflammation. The authors reported that isoorientin inhibited the nuclear translocation of p65 subunit of NF-*κ*B induced by LPS, cylooxygenase-2 (COX-2), tumor necrosis factor-*α* (TNF-*α*), interleukin-6 (IL-6), 5-lipoxygenase (5-LOX), and interleukin-1-*β* (IL-1-*β*) and induced glutathione S-transferase and catalase. D. Beghelli et al. reported immunomodulatory activities for the extract of *Boswellia serrata* (BS), a traditional medicinal plant, which has in vitro antioxidant activity due to its significant phenolic content. In addition, different extracts (oleogum and aqueous) were reported to have different effects on regulatory T cells (Treg) and T helper IL-17- (Th17-) producing cells.

Two studies in this issue evaluated the potential antioxidant and anti-inflammatory effects of natural compounds in humans in relation to metabolic profiles. In patients with metabolic syndrome, the consumption of Goji berry for 45 days improved waist circumference, transaminases, lipid profile, lipid peroxidation, glutathione, and catalase, but no effects were observed on inflammatory markers, including C-reactive protein and TNF-*α* (S. Zanchet et al.). In a pilot study in overweight/obese women, the consumption of an orange juice rich in anthocyanins over a period of 12 weeks improved LDL-cholesterol but had no significant effects on body weight, blood pressure, insulin resistance, antioxidants, and inflammatory status (E. Azzini et al.). Moreover, the authors suggested that different grades of obesity resulted in different changes in inflammation biomarkers after orange juice consumption.

Peluso and coworkers performed an initial study to evaluate the rejection by the European Food Safety Administration (EFSA) and the Supplement Information Expert Committee (DSI EC) regarding oxidative stress-related bioremedial claims of tea and more specifically green tea extract (GTE). Their investigation based on the peroxidation of leukocytes index ratio (PLIR) and ferric reducing antioxidant potential (FRAP) largely corroborated the EFSA and DSI EC assessment. Albeit that the concentration-dependent prooxidant capacity of most low molecular weight antioxidants has been known for a long time and oxidative stress-related health claims should always be evaluated with care, further investigations on tea and tea-related extracts should be performed over a wide concentration range and in intact organisms in order to include potential potentiating or attenuating cofactors. Finally, the authors once more confirm the essentiality of uric acid in the first line defence against ROS.

I. Marrocco et al. reviewed the biomarkers of oxidative stress in humans and showed that significant methodological bias must be taken into account when interpreting data from the measurement of reactive species in leukocytes and platelets by flow cytometry, from the evaluation of markers based on ROS-induced modifications, from assays regarding the enzymatic players of redox status, and from the measurement of the total antioxidant capacity of human body fluids.

Although it has been suggested that this bias in each method could be overcome by the evaluation of oxidative stress by using more than one criterion, it must be taken into consideration that the clinical significance of any biomarker of oxidative stress in humans must come from a critical analysis of potential bias and that the choice of marker must be considered in the global index, which should be dictated by the subjects' characteristics and by the study aim and design.

We hope that this issue will stimulate significant discussion on the potential confounding of oxidative stress evaluation and on the clinical significance of oxidative stress biomarkers.

